# Lipid Nanoparticles for Ocular Gene Delivery

**DOI:** 10.3390/jfb6020379

**Published:** 2015-06-08

**Authors:** Yuhong Wang, Ammaji Rajala, Raju V. S. Rajala

**Affiliations:** 1 Dean A. McGee Eye Institute, Oklahoma City, OK 73104, USA; E-Mails: yuhong-wang@ouhsc.edu (Y.W.); ammaji-rajala@ouhsc.edu (A.R.); 2 Department of Ophthalmology, College of Medicine, University of Oklahoma, Oklahoma City, OK 73014, USA; 3 Department of Physiology and Harold Hamm Diabetes Center, University of Oklahoma Health Sciences Center, Oklahoma City, OK 73014, USA

**Keywords:** gene therapy, non-viral vectors, lipid nanoparticles, solid lipid nanoparticles, liposomes

## Abstract

Lipids contain hydrocarbons and are the building blocks of cells. Lipids can naturally form themselves into nano-films and nano-structures, micelles, reverse micelles, and liposomes. Micelles or reverse micelles are monolayer structures, whereas liposomes are bilayer structures. Liposomes have been recognized as carriers for drug delivery. Solid lipid nanoparticles and lipoplex (liposome-polycation-DNA complex), also called lipid nanoparticles, are currently used to deliver drugs and genes to ocular tissues. A solid lipid nanoparticle (SLN) is typically spherical, and possesses a solid lipid core matrix that can solubilize lipophilic molecules. The lipid nanoparticle, called the liposome protamine/DNA lipoplex (LPD), is electrostatically assembled from cationic liposomes and an anionic protamine-DNA complex. The LPD nanoparticles contain a highly condensed DNA core surrounded by lipid bilayers. SLNs are extensively used to deliver drugs to the cornea. LPD nanoparticles are used to target the retina. Age-related macular degeneration, retinitis pigmentosa, and diabetic retinopathy are the most common retinal diseases in humans. There have also been promising results achieved recently with LPD nanoparticles to deliver functional genes and micro RNA to treat retinal diseases. Here, we review recent advances in ocular drug and gene delivery employing lipid nanoparticles.

## 1. Introduction

The eye is made up of many components, and therapeutic agents could be easily applied to the anterior part of the eye. However, it is difficult to administer these agents to the posterior part of the eye. Intravitreal or subretinal routes are the only means of targeting agents to the posterior area of the eye. The eye is one of the sensory organs of the body, and frequent administration of drugs to the eye is undesirable. Therefore, gene therapy would be an ideal way to provide sustained gene expression that could overcome these limitations. The eyes have been early targets for gene therapy because they are small—that is, they require relatively little active dose—they are self-contained, and because the tools of eye surgery have advanced enough to make these treatments possible. The eye offers an excellent target for gene therapy studies, it is easily accessible and relatively immune privileged. If we inject any drug or gene systemically, the drug or gene must then cross the blood retinal barrier (BRB). To our knowledge, most of the successful gene therapy trials use local administration of drug(s)/gene(s) into the eye. 

## 2. Uses and Advantages of Nanoparticles in Medicine

Nanoparticles play important roles in the diagnosis of disease, delivery of drugs to target tissue, research into the organization of DNA, drug-mediated apoptosis of cancer cells, studies of the pharmacological efficiency of drugs, and tissue engineering. Their size and surface characteristics enable us to alter nanoparticle properties to allow for continuous discharge of drugs during transport and release at a defined location. Choosing the appropriate matrix is vital to drug delivery. Modifying the surface properties of nanoparticles will help to clear the drug from the patient’s body with significantly fewer side effects. 

These particles are currently conjugated with either drugs or genes and administered through several avenues, including the oral, nasal, intra-ocular, and arterial routes. Researchers are exploring the use of various polymers to conjugate drugs and genes to enhance therapeutic benefits while minimizing adverse effects. 

Nanoparticles for gene therapy are broadly classified into three groups, metal-based nanoparticles, lipid based-nanoparticles, and polymer-based nanoparticles. These particles are different in size, charge, shape, and structure, and have their own modes of delivering cargo into cells and assimilating the cargo into the genetic machinery for gene expression [1,2,3,4]. Compacted DNA nanoparticles formulated with polyethylene glycol-substituted polylysine have been used for gene therapy in mouse models of eye diseases [5,6,7,8]. Solid lipid nanoparticles (SLNs) and nanostructured lipid carriers (NLCs) have been developed to improve the ocular delivery of acyclovir into excised corneal tissue [9]. 

A solid lipid nanoparticle is typically spherical, with an average diameter between 10 and 1000 nm, and possesses a solid lipid core matrix that can solubilize lipophilic molecules. The lipid core is stabilized by surfactants; the lipid component may be a triglyceride, diglyceride, monoglyceride, fatty acid, steroid, or wax. The lipid nanoparticle, called the liposome protamine/DNA lipoplex (LPD), is electrostatically assembled from cationic liposomes and an anionic complex of protamine and DNA. The LPD nanoparticles contain a highly condensed DNA core, surrounded by lipid bilayers with an average size of ~100 nm. Lipid nanoparticles have also been used to improve the efficiency of siRNA delivery in RPE cells and a laser-induced rat model for the treatment of choroidal neovascularization [10]. 

Methazolamide (MTA) is an anti-glaucoma drug; however, systemic administration produces side effects, while providing insufficient ocular therapeutic concentrations [11]. Solid lipid nanoparticles containing MTA have been shown to have higher therapeutic efficacy at low doses with more prolonged effects than those of the drug solution itself [11]. Lipid nanoparticles have also been shown to be feasible for the ocular delivery of anti-inflammatory drugs [12]. 

Since the 1990s, solid lipid nanoparticles have been examined as potential drug carrier systems. SLNs do not show bio-toxicity, as they are prepared from physiological lipids, and are especially useful in ocular drug delivery, as they enhance the corneal absorption of drugs and improve the ocular bioavailability of both hydrophilic and lipophilic drugs [13]. Cyclosporine is commonly prescribed for chronic dry eye, caused by inflammation, and cyclosporine A-loaded solid lipid nanoparticles have been shown to improve drug efficacy when administered to rabbit eyes [14,15]. 

Liquid lipid has also been incorporated into lipid nanoparticles to enhance ocular drug delivery [16]. These particles have been tested on human corneal epithelial cell lines and rabbit corneas [16]. The liquid lipid incorporation has been shown to improve the ocular retention and penetration of therapeutics [16]. Surface-modified solid lipid nanoparticles have been shown to provide an efficient way of improving the ocular bioavailability of drugs to bioengineered human corneas [17]. Solid lipid nanoparticles have also been used for retinal gene therapy and to study intracellular trafficking in RPE cells [18]. Solid lipid nanoparticles and lipid nanoparticles have been extensively reviewed and described in detailed in recently published articles [19,20]. 

The majority of solid lipid nanoparticles have been used to deliver drugs to the cornea [9,13,16,17,21,22]. We recently formulated a novel lipid nanoparticle, and examined its efficiency and delivery of genes and microRNA to the retina [23,24]. LPD has been used to successfully deliver the vascular endothelial growth factor gene into mesenchymal stem cells [25]. In this article, we review several important aspects of lipid nanoparticles, including their formulation, mechanism of internalization, cell-specific expression, and barriers that affect gene expression. 

## 3. Gene Therapy and Viral Vectors

The success of gene therapy relies on the development of efficient, non-toxic gene carriers that can encapsulate and deliver foreign genetic materials into specific cell types [26]. Gene therapy carriers can be classified into two groups, viral and non-viral gene delivery systems. Although viral vectors, such as adeno-associated virus (AAV), have attractive features, particularly their high gene transduction capability, they face biosafety issues, especially innate and immune barriers [27], toxicity [28], and potential recombination of or complementation [29] to vector delivery. The size of viral vectors, which restricts the insertion of genes to <5 kb, is another limitation [30]. Table 1 lists various viral and non-viral carriers. All viral vectors have been used to deliver functional genes to the retina whereas non-viral vectors have been used to deliver both drugs (liposome nanoparticles and solid lipid nanoparticles) and genes (solid lipid nanoparticles, LPD/lipoplexes and CK30-PEG) to the retina. 

**Table 1:** Viral and non-viral delivery systems for ocular gene delivery.

Vector	Carrier	Delivery	Ref.
Virus	AAV	Local/systemic	[31,32,33,34,35]
	Adenovirus	Local	[36]
	Baculovirus	Local	[37,38]
	Lentivirus	Local	[39]
Non-virus	Liposome nanoparticles	Local	[40,41,42,43]
	Solid lipid nanoparticles	Local	[9,13,16,17,21,22]
	LPD/lipoplexes	Local	[23,24,44]
	CK30-PEG	Local	[5,6,7,8]

Despite rapid advances in gene therapy during the last two decades, major obstacles to clinical applications for human diseases still exist. These impediments include immune response, vector toxicity, and the lack of sustained therapeutic gene expression. Therefore, new strategies are needed to achieve safe and effective gene therapy. The ideal vector should have low antigenic potential, high capacity to accommodate genetic material, high transduction efficiency, controlled and targeted transgene expression, and reasonable expense and safety for both the patients and the environment. These desired features led researchers to focus on non-viral vectors as an alternative to viral vectors. 

## 4. Lipid-Based Nanoparticles

**Figure 1:**
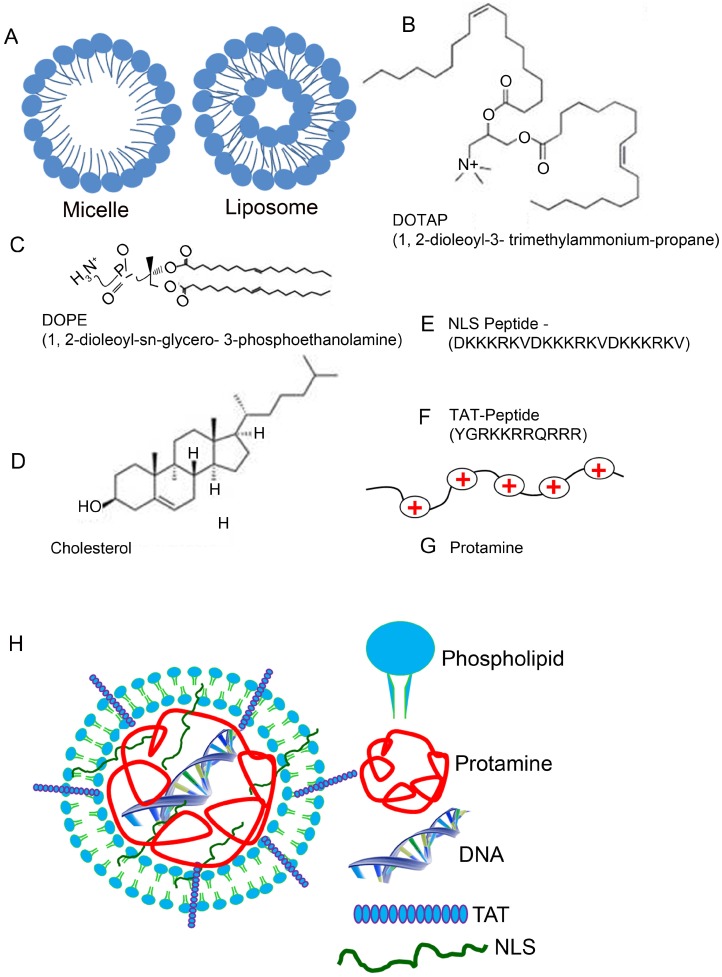
Lipid, peptide, and protein components of the lipid nanoparticle. The monolayer structures are called micelles, whereas the lipid bilayer structures are called liposomes (**A**). Chemical structures of DOTAP (**B**), DOPE (**C**), and Cholesterol (**D**). NLS (**E**) and TAT (**F**) peptide sequences and protamine (small, arginine-rich, nuclear protein) (**G**) are also presented. DOTAP, 1, 2-dioleoyl-3-trimethylammonium-propane, DOPE, 1, 2-dioleoyl-sn-glycero-3-phosphoethanolamine; NLS, nuclear localization signal; TAT, transactivator of transcription. Formulation of a peptide-based lipid nanoparticle (**H**). Peptide-based nanoparticles can be formulated by mixing liposome, protamine, DNA, TAT, and NLS. TAT, transactivator of transcription; NLS, nuclear localization signal.

The main constituent of lipid nanoparticles is the liposome. A liposome is a spherical vesicle of a lamellar phase of the lipid bilayer. The liposome can be used as a transport vehicle to send nutrients and drugs into the body [45,46,47]. One can prepare these liposomes through disruption of biological membranes by sonication, a process of sending sound waves to disturb particles in a solution. Lipids can naturally form themselves into nano-films and nano-structures, called micelles, reverse micelles, and liposomes [20,48]. The monolayer structures are called micelles or reverse micelles, whereas the lipid bilayer structures are called liposomes (Figure 1A). In the lipid bilayer, phospholipids are principal lipids, which are amphiphilic molecules with hydrophilic (*water-loving*, polar) and lipophilic (*fat-loving*) properties, sometimes described as having hydrophobic tails and hydrophilic heads. Therefore, liposomes are artificial phospholipid bilayers; as a result, liposomes have biocompatible characteristics [49,50]. This biocompatibility accounts for their most important advantages as drug carriers, (1) liposomes have almost no toxicity and low antigenicity; (2) liposomes can be biodegraded and metabolized *in vivo*, and (3) liposomal properties, such as membrane permeability, can be controlled to some extent [51,52,53]. Remarkably, liposomes can entrap and protect drug molecules or nucleic acids on the journey to the target site [54]. 

When nucleic acids, molecules, or drugs are enclosed in a lipid-based coating, they have a lower degradation rates than do molecules without a lipid coating. Such enclosure also increases the likelihood of endocytosis and uptake of nucleic acids or drugs into cells [4,20,55]. These desired features led researchers to focus on non-viral vectors as an alternative to viral vectors. The non-viral vectors include polymers like polyethylenimine (PEI) [56] and poly L-lysine (PLL) [57], peptides, liposomes (tiny fat-like particles) [58], and liposomes-protamine-DNA (LPD) complexes [59,60]. However, the current non-viral vectors cannot achieve tissue-specific or cell-specific sustained gene expression, nor eliminate the unwanted and harmful effects on other cells. 

The use of lipid nanoparticles as part of a system delivering drugs and genes to the retina has been attempted [44]. We recently developed an artificial virus, an LPD nanoparticle in combination with nuclear localization signaling (NLS) [61] peptide and transactivator of transcription (TAT) peptide [62], to produce efficient, cell-specific gene delivery to eye tissues, with sustained gene expression. The key to our success arises from three unique features, (1) the use of biocompatible lipid molecules to pack DNA and the biocompatible protamine molecules into the nanoparticles; (2) the integration of cell-penetrating and nuclei-targeting peptides into the nanoparticles, to improve the efficiency of gene transfer and the subsequent lasting gene expression; and (3) the use of a DNA that carries the target gene, and also bears a unique promoter to achieve cell-specific gene expression. 

## 5. Composition of Lipid Nanoparticles

Liposomes were first identified in 1965 [63], and were successfully applied as cationic liposome complexes via intravenous DNA delivery into adult mice in 1993. Since then, liposomes have been successful and widely applied in nanotechnology [64] and in various medical fields [23,24,61,65,66]. One approach for more successful nanoparticle gene therapy is the liposome protamine/DNA lipoplex (LPD), which is applied as a two-step packaging technology employing a multilayering method [61,67]. First, the DNA is packaged into a condensed core via electrostatic interactions with protamine, and various peptides (NLS and TAT) and the plasmid DNA (pDNA) are mixed at various weight ratios (Figure 1). Then, the liposomes, consisting of a cationic lipid DOTAP (1,2-dioleoyl-3-trimethylammonium-propane), a neutral “helper” lipid DOPE (1,2-dioleoyl-sn-glycero-3-phosphoethanolamine) and neutral cholesterol, are added so that the positively charged DOTAP/DOPE/Chol liposome can form a complex with the negative protamine/DNA particles, leading to the formation of LPD nanoparticles (Figure 1B–G). The negatively charged DNA is complexed with protamine, an arginine-rich, positively charged nuclear protein that replaces histone late in the haploid phase of spermatogenesis, and is essential for sperm head condensation and DNA stabilization. The advantage of adding protamine to DNA is that protamine condenses the DNA and the subsequent mixing of the protamine/DNA complex to cationic liposomes, producing a small nanoparticle. Another advantage of protamine is that the encapsulated DNA is protected from nuclease degradation [23,59]. Inclusion of protamine in solid lipid nanoparticles (SLN) has previously been shown to yield a six-fold increase in the transfection of SLN in retinal cells, due to the presence of a nuclear localization signal [68]. 

## 6. Transfer Mechanism of LPD Nanoparticles into Cells

Successful gene delivery systems have their own transfer mechanisms into the cell. Viral vectors have the advantage in cellular entry, because they bind to the cellular receptors and co-receptors, which help them to internalize and traffic to the nucleus [69,70,71,72,73]. In contrast, cationic liposomes take advantage of biocompatible characteristics and are widely used to transfect DNA into cells in culture and *in vivo*, since the formation of cationic lipid-DNA complexes can facilitate the association with the cell membrane and allow the complex to enter the cell through the endocytotic pathway [4,58,74]. The complex is internalized into an endosome, which will destabilize the endosome membrane and result in a flip-flop of anionic lipids that are mainly on the cytoplasmic side of the membrane. The anionic lipids will then diffuse into the complex and form charge-neutralized ion pairs with cationic lipids. This displaces the DNA from the complex and allows DNA to enter into the cytoplasm [4,74,75]. Protamine in the solid lipid nanoparticles has been reported to shift the internalization mechanism from caveolae/raft-mediated to clathrin-mediated endocytosis [68]. Some researchers also proposed that LPD nanoparticles could use two different endocytosis pathways, macropinocytosis and clathrin-mediated endocytosis [58]. In the final analysis, liposomes depend on continually improving the formulation of the nanoparticles’ coating and DNA design to increase the transfection efficiency [76,77]. The mechanisms by which peptide-modified liposome protamine/DNA lipoplex (LPD) nanoparticles improve transfer efficiency is charge-ratio-dependent and dose-dependent *in vivo*, and these mechanisms provide their own unique approaches to improve transfer efficiency [23,59,61]. 

## 7. Cellular Barriers in the Internalization of Lipid Nanoparticles

**Figure 2:**
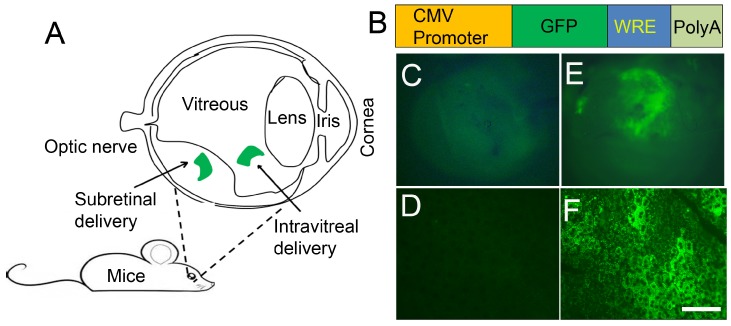
LPD-mediated gene delivery into the retina. Schematic illustration of the eye and route of administration. The most commonly used and preferred mode of administration to retinal layers is subretinal (**A**). Generation of green fluorescent protein construct under the control of CMV promoter (**B**). CMV, cytomegalovirus; GFP, green fluorescent protein; WRE, posttranscriptional regulatory element from the woodchuck hepatitis virus; PolyA, polyadenylation sequence; increases the stability of the molecule. Using BalbC mice, we injected the cDNA construct subretinally into one eye. LPD was complexed with CMV-GFP-WRE-PolyA construct. The other eye was injected with LPD, with a control vector without GFP. Seventy-two hours later, eyes were removed and examined for GFP expression under inverted fluorescence microscopy. GFP expression is clearly seen in the GFP-injected eye (**E**), but not in the control eye (**C**). Whole RPE flat mounts were prepared and examined for GFP expression under inverted fluorescence microscopy. GFP expression is seen in the GFP-injected eye (**F**), but not in the control eye (**D**). Scale bar, 20 µm.

DNA packed into liposomes must overcome biological barriers before it can be integrated into the genome. These barriers are the cellular membrane, the nuclear membrane, and chromosomal integrity. Cell targeting and cell-internalization peptides have been extensively studied and used for efficient drug delivery and for image analysis [61]. Arginine-rich (RNA-binding, DNA-binding, and polyarginine) cell-permeable peptides have been shown to cross the cellular barrier [62]. Nuclear localization peptide of the SV40 T large antigen has been shown to promote high LPD-mediated transfection efficiency [23,24,61,78]. In designing our recently formulated lipid nanoparticle, we used a nuclear localization peptide derived from SV40 T antigen (DKKKRKVDKKKRKVDKKKRKV), and another peptide derived from human immunodeficiency virus transactivator of transcription (TAT; YGRKKRRQRRR) peptide [79,80,81,82]. The TAT-fusions have been shown to cross the blood–brain barrier [81]. A combination of these two peptides resulted in a high level of sustained gene expression *in vivo* (Figure 2) [23]. The TAT-peptide belongs to an arginine-rich family of peptides, which is an abundant source of membrane-permeable peptides that have potential as carriers for intracellular protein delivery [54,67]. Even with the omission of TAT-peptide, LPD nanoparticles were able to mediate gene delivery [24]. 

The cell-penetrating peptides (CPPs) are short peptides that facilitate cellular uptake of various molecular cargo [63,64]. In 1988, the first CPP was sequenced from an HIV-1-encoded cell-penetrating transactivator of transcription (TAT) peptide, and delivered efficiently through cell membranes; TAT has been widely applied since then [83,84,85]. The TAT mechanism of action is still poorly understood, but we do know that this TAT may possess a common internalization mechanism that is ubiquitous to arginine-rich peptides. However, the mechanism is not explained by either adsorptive-mediated endocytosis or by receptor-mediated endocytosis [62,86,87].

## 8. LPD Nanoparticle-Mediated Delivery of Genes to Eye Tissues

In the eye, the photochemical 11-*cis-*retinal allows the visual pigment rhodopsin to absorb light in the visible range. Without the photochemical, we lose the ability to see light [88]. Pre-clinical studies with viral vectors demonstrated restoration of vision upon gene transfer into retinal cells in mice and dogs [31,32,33,34]. In clinical trials, three independent groups reported vision improvements upon the viral-mediated delivery of the Rpe65 gene in patients with Rpe65-associated Leber’s congenital amaurosis (LCA) [89,90,91]. A mouse model lacking the Rpe65 gene has been commonly used for gene therapy studies [5,92,93,94]. 

Retinal pigment epithelium protein 65 (Rpe65) is the key enzyme in regulating the availability of photochemicals; a deficiency in this gene results in a blinding eye disease. We showed for the first time that LPD promotes efficient delivery in a cell specific-manner and long-term expression of the Rpe65 gene in mice lacking Rpe65 protein, leading to *in vivo* correction of blindness [23]. The efficacy of this method of restoring vision is comparable to AAV [93] and lentiviral [39] gene transfer of the Rpe65 gene to Rpe65 knockout mice. Our recently published data suggest that we successfully applied LPD to deliver miRNA-184 to the retina, to repress Wnt-mediated ischemia-induced neovascularization [24]. Thus, LPD nanoparticles could provide a promising, efficient, non-viral method of gene delivery with clinical applications in the treatment of eye disease. 

## 9. Cell-Specific Delivery of LPD Nanoparticles

One disadvantage of nanoparticles could be cell specificity. Often, delivery and expression of genes in unwanted cells may lead to adverse or off target effects. We recently achieved specificity by cloning the genes under the control of cell-specific promoters [23]. VMD2-promoter specifically targets LPD to RPE cells, whereas rod opsin promoter specifically drives the expression into rod photoreceptor cells [23]. These studies suggest that other retinal cell specific promoters, such as cone opsin (cone), Thy1 (ganglion cell), and glial fibrillary acidic protein (Müller cells) could be used to achieve cell specificity in conjunction with LPD. The cytomegalovirus (CMV) promoter is widely used, due to its ability to induce protein expression in varied cell types [1,23]. Interestingly, our recent study suggests that CMV promoter exclusively drives expression in retinal pigment epithelial cells [23]. These features make lipid nanoparticles ideal for gene or drug delivery to ocular tissues. 

## 10. Conclusions

Many unique genes have been associated with major retinal diseases, such as retinitis pigmentosa (RP), Leber’s congenital amaurosis (LCA), and Stargardt disease [65,69,95]. Until now, Rpe65 defection-induced LCA has been most extensively researched retinal disease. LCA-Rpe65 gene therapy is an example of successful, innovative, translational research. Further studies are needed to determine how retinal gene therapy can be improved [96,97]. The LPD is modified with cell-penetrating peptide and NLS peptide, and carries DNA capable of cell-specific gene expression. Our recent studies suggest that LPD promotes efficient and lasting gene expression *in vivo* without any corresponding inflammatory response [23]. 

The LPD system could be a promising non-viral gene delivery vector yielding long-term expression and lasting gene transfer efficiency, making it a favorable gene carrier for future applications for eye cell-based therapies. The advantage is that this system allows us to simultaneously introduce multiple biomolecules to turn on the defective signaling pathway *in vivo*. Thus far, non-viral vectors have traditionally been acknowledged as safer. However, non-viral vectors present their own difficulties, with low gene expression efficiency and short transient expression. Recently, the peptide-modified liposome protamine/DNA lipoplex (LPD) nanoparticle has demonstrated the potential to overcome these barriers. 

Based on the successful gene therapy of Rpe65 in peptide-modified LPD nanoparticles, the optimization of liposome nanoparticle formulations is safe and efficient. Improvements in gene expression are key to the further development of liposomal nanoparticle technology for retinal gene therapy. The development of modified and safe delivery systems to optimize transfection efficiency will be a critical step toward clinical trials for human gene therapy. Thus, these new peptide-modified LPD nanoparticles open avenues to investigate and develop highly efficient liposome nanoparticles that can overcome the shortcomings of other viral vectors in the treatment of ocular diseases. 

These peptide-modified LPD have many advantages for future clinical applications. First, liposome nanoparticles are able to deliver large molecular cargo. Second, the optimization of peptide-modified LPD nanoparticles allows multiple mutant genes to be simultaneously co-delivered to one vector. Third, peptide-modified LPD formulations are more biocompatible and safe. On the whole, a successful delivery formulation for gene therapy should encapsulate and protect the nucleic acid materials, escape endosomal degradation, and reach the specific target site. These new peptide-modified LPD nanoparticles offer new hope for gene therapy for ocular and other related diseases. 
